# The Influence of Auditory Attention on Rhythmic Speech Tracking: Implications for Studies of Unresponsive Patients

**DOI:** 10.3389/fnhum.2021.702768

**Published:** 2021-08-11

**Authors:** Rodika Sokoliuk, Giulio Degano, Lucia Melloni, Uta Noppeney, Damian Cruse

**Affiliations:** ^1^School of Psychology, University of Birmingham, Birmingham, United Kingdom; ^2^Centre for Human Brain Health, University of Birmingham, Birmingham, United Kingdom; ^3^Brain and Language Lab, Department of Psychology, Faculty of Psychology and Educational Sciences, University of Geneva, Geneva, Switzerland; ^4^Max Planck Institute for Empirical Aesthetics, Frankfurt, Germany; ^5^Department of Neurology, New York University, New York City, NY, United States; ^6^Donders Centre for Cognitive Neuroimaging, Nijmegen, Netherlands; ^7^Department of Biophysics, Radboud University, Nijmegen, Netherlands

**Keywords:** cortical tracking, brain oscillations, EEG, attention, speech tracking, unresponsive patients

## Abstract

Language comprehension relies on integrating words into progressively more complex structures, like phrases and sentences. This hierarchical structure-building is reflected in rhythmic neural activity across multiple timescales in E/MEG in healthy, awake participants. However, recent studies have shown evidence for this “cortical tracking” of higher-level linguistic structures also in a proportion of unresponsive patients. What does this tell us about these patients’ residual levels of cognition and consciousness? Must the listener direct their attention toward higher level speech structures to exhibit cortical tracking, and would selective attention across levels of the hierarchy influence the expression of these rhythms? We investigated these questions in an EEG study of 72 healthy human volunteers listening to streams of monosyllabic isochronous English words that were either unrelated (scrambled condition) or composed of four-word-sequences building meaningful sentences (sentential condition). Importantly, there were no physical cues between four-word-sentences. Rather, boundaries were marked by syntactic structure and thematic role assignment. Participants were divided into three attention groups: from passive listening (passive group) to attending to individual words (word group) or sentences (sentence group). The passive and word groups were initially naïve to the sentential stimulus structure, while the sentence group was not. We found significant tracking at word- and sentence rate across all three groups, with sentence tracking linked to left middle temporal gyrus and right superior temporal gyrus. Goal-directed attention to words did not enhance word-rate-tracking, suggesting that word tracking here reflects largely automatic mechanisms, as was shown for tracking at the syllable-rate before. Importantly, goal-directed attention to sentences relative to words significantly increased sentence-rate-tracking over left inferior frontal gyrus. This attentional modulation of rhythmic EEG activity at the sentential rate highlights the role of attention in integrating individual words into complex linguistic structures. Nevertheless, given the presence of high-level cortical tracking under conditions of lower attentional effort, our findings underline the suitability of the paradigm in its clinical application in patients after brain injury. The neural dissociation between passive tracking of sentences and directed attention to sentences provides a potential means to further characterise the cognitive state of each unresponsive patient.

## Introduction

A growing body of evidence indicates that a proportion of patients who are unresponsive as a result of severe brain injuries nevertheless produce patterns of brain activity that are indicative of language comprehension (Coleman et al., 2009; Braiman et al., 2018; Gui et al., 2020; Sokoliuk et al., 2021). Due to the potential clinical impact of concluding that a behaviourally unresponsive patient comprehends what is said to them, it is vital that we accurately characterise the precise cognitive mechanisms for which we have evidence, as well as their link to the patients’ levels of consciousness.

Conscious speech comprehension involves a hierarchy of progressively more complex neural processes, from acoustics through to meaning. For example, reading a book on public transport can be challenging, especially if people around us talk about their personal life, or the latest gossip in the neighbourhood, leaving us unwillingly trapped in their conversations. Disconnecting becomes easier if we do not understand the language. In both cases, however, our auditory neurons follow the rhythm of individual syllables (4–8 Hz; Ghitza, 2013), suggested to reflect entrainment of underlying brain oscillations to acoustic features of speech (Poeppel, 2003; Ghitza, 2011, 2012, 2013; Giraud and Poeppel, 2012), boosting its intelligibility (Luo and Poeppel, 2007; Elliott and Theunissen, 2009; Zoefel and VanRullen, 2015, 2016).

Using natural speech stimuli, recent studies showed that their envelopes are tracked by the brain activity of healthy participants with high temporal precision (e.g., Lalor and Foxe, 2010; Ding and Simon, 2013) and that the precision of this envelope tracking predicts recognition of the speech signal (Ding and Simon, 2013).

In addition to the relatively rapidly changing acoustic envelope of syllables in an acoustic speech stimulus, language comprehension is based on slower rhythms, reflecting the syntacto-semantic link between syllables. For instance, the mono-syllabic words “sharp-knife” are in themselves informative but also build a meaningful phrase (Chomsky, 1957). In the same way, but on a larger scale, we know when a sentence begins and ends based on the meaning of individual syllables/words and their syntax. Importantly, acoustic cues, like gaps separating phrases or sentences, are not necessary. Slow neural rhythms in speech comprehension have been observed with magnetoencephalography (MEG; Ding et al., 2016) and surface and intracranial electroencephalography (iEEG, Ding et al., 2016; EEG, Ding et al., 2017). In one MEG study, participants listened to mono-syllabic isochronous words, played consecutively. These were either unintelligible syllables/unrelated words (scrambled condition; e.g., “cold-eat-cell-dog…”) or sequences of four syllables/words building a meaningful sentence (sentential condition; e.g., “sharp-knife-cuts-meat…”). Both conditions led to a peak at the syllable/word frequency in the power spectra – i.e., tracking – but crucially, tracking at the rate of the phrases (“sharp-knife”) and sentences (“sharp-knife-cuts-meat”) was observed only in the sentential condition, despite there being no acoustic stimulus changes at those rates. Importantly, these peaks were only found when participants understood the language (Ding et al., 2016) and were awake (Makov et al., 2017). Therefore, it has been suggested that these slower brain oscillations might reflect speech comprehension (Ding et al., 2016, 2017; Makov et al., 2017). Throughout this article, we describe this effect as “tracking,” rather than “entrainment,” which would impose phase alignment between an exogenous signal (i.e., rhythmic auditory stimulus) and an underlying oscillatory process. Since the existence of this potential underlying oscillatory process cannot be proved with this dataset, we decided to use the term “tracking” to avoid this ambiguity (see also Obleser and Kayser, 2019).

Whether or not tracking of higher-level linguistic structures such as phrases and sentences reflects conscious comprehension of speech also carries clinical relevance, as this paradigm has recently been shown successful in predicting the outcome of chronic (Gui et al., 2020) and acute unresponsive patients (Sokoliuk et al., 2021). Although language comprehension was not explicitly tested in any of the previous studies using this paradigm (e.g., *via* comprehension questions or subsequent memory), a recent study shows evidence for awareness being a requirement for grouping visually presented individual words into larger linguistic units like phrases or sentences (Rabagliati et al., 2018). While the stimuli used here, as well as in the previous studies mentioned above, are different from natural speech, as their linguistic structures follow an exact temporal pattern, they appear to be ideal to identify such high-level language processing in a listener. However, the link between evidence for language comprehension and the state of consciousness and cognition in patients is unclear. To date, all studies using this paradigm in healthy participants also informed the participants about the sentential stimulus structure and almost all studies involved an active task that required sentence comprehension (see Gui et al., 2020, for a study that used a passive listening condition, followed by a subsequent memory task). Active tasks have been used with some success in disorders of consciousness, by investigating covert command following, for instance (Owen et al., 2006; Monti et al., 2010; Cruse et al., 2011). However, it has been argued previously (Cruse et al., 2011; Sokoliuk et al., 2021) that these tasks exclude those with severe cognitive deficits and that a higher number of patients with residual consciousness could be identified by applying entirely passive paradigms. An important point to clarify for the cortical tracking paradigm is therefore: what is the influence of the active task on the strength of higher-level tracking? Would sentence tracking also be present in naïve participants? What is the influence of goal-directed attention on the acoustic and linguistic tracking?

**FIGURE 1:**
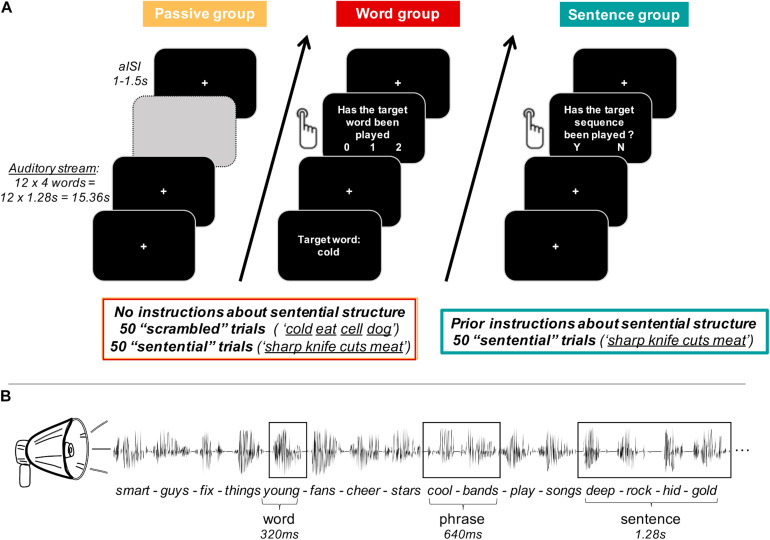
Experimental paradigm. **(A)** Participants were divided into three groups of 24 participants each. For every group, auditory stimuli of each trial were presented as a continuous stream of 12 four-word-sequences, which were built by concatenating isochronous single words of 320 ms length each. The passive group (orange) was naïve to the sentential structure of the stimulus material and passively listened to the auditory stream. The word group (red) was also naïve to the sentential structure and had an active task based on individual target words. After each trial, participants were asked whether the target word had been presented 0, 1, or 2 times. Both the passive and the word group were exposed to stimuli of the scrambled condition (unrelated words) and the sentential condition (four consecutive words built a meaningful sentence). The sentence group (turquoise) was instructed about the sentential structure in the stimulus material and was only listening to the sentential condition. Participants were asked to report via button press after each trial, whether a grammatically incorrect four-word-sequence had been played in the respective trial. For each participant group, individual trials were separated by an asynchronous inter-stimulus-interval (aISI) of 1–1.5 s. (Greyed out field only serves visualisation purposes of this figure.) **(B)** Example of four four-word-sentences (sentential condition only) to illustrate temporal properties of individual linguistic structures.

We investigated these questions in an EEG study on 72 healthy human volunteers, listening to mono-syllabic isochronous English words (cf. Figure 1). Similar to the previous studies, in the sentential condition, four consecutive words built a meaningful sentence (*adjective-noun-verb-noun*), whereas in the scrambled condition, successive words were unrelated (e.g., *adjective-verb-noun-noun*). Participants were either passively listening (passive group), attending to individual words (word group), or to sentences (sentence group; comparable with previous studies). While the passive and word groups were naïve to the sentential stimulus structure, the sentence group was instructed about it prior to the experiment. This combination of stimulus and task manipulations allowed us to orthogonally isolate the roles of attention on neural tracking of low-level (words) and high-level (sentences) features. Characterising the cognitive processes reflected in higher-level tracking in healthy participants in this way provides clearer insights into the state of cognition and consciousness preserved by each given unresponsive patient.

## Materials and Methods

### Participants

We recorded behavioural and EEG data of 72 healthy human volunteers (median age: 22, range: 18–33; 42 females). Prior to any data acquisition and prior to recruitment of participants, a randomised list was created, assigning 48 planned participants to either the “word” or the “passive” participant group. Data collection of the remaining 24 participants was completed after data collection of the first two groups and therefore all 24 recruited participants were assigned to the “sentence” participant group. We used the same self-reported inclusion criteria for all participants, who reported to be monolingual English speakers, between 18 and 35 years old, right-handed, with no history of epilepsy, and no diagnosis of dyslexia. Participants received either course credits or a monetary compensation for participation. The experimental procedures were approved by the Ethical Review Committee of the University of Birmingham (ERN_15-1367AP3) and conformed to the Declaration of Helsinki. All participants gave written informed consent prior to participation in the study. Data of five participants were excluded from the analysis after visual inspection because of excessive artefacts in the EEG signal (e.g., muscular artefacts) and two participants in the sentence group because of poor behavioural performance (≤50%), resulting in total in 20, 23, and 22 participants for the passive group, the word group, and the sentence group, respectively.

### Stimuli

We constructed a total of 288 mono-syllabic English words using the male voice of the Apple synthesiser (Macintalk, voice Alex; Apple MacBook Pro Third generation), and these words were segmented using Audacity software version 2.1. Importantly, words were isochronous, of 320 ms in length, which resulted in a presentation frequency of 3.125 Hz for the word rate, 1.56 Hz for the phrase rate, and 0.78 Hz for the sentence rate. The words included 144 nouns, 72 adjectives, and 72 verbs (full word list is available on OSF under the following link: https://osf.io/8pu4a/). For the sentential condition, a total of 72 four-word-sentences were constructed, conforming to the syntactic structure: adjective – noun – verb – noun. Each four-word-sentence was played a minimum of eight and a maximum of nine times per participant throughout the experiment. The order with which they were presented was randomly chosen on a trial-by-trial basis, avoiding occurrence of the same four-word-sentence more than once per trial. For the sentence group only, 10% of the trials contained target sequences, which were grammatically incorrect and either followed the order “noun – noun – adjective – verb” or “adjective – verb – noun – noun.”

In the scrambled condition, every trial consisted of 12 four-word-sequences. Each contained four randomly chosen individual words of a given word category, i.e., two nouns, one adjective, and one verb from the total of 288 words. To ensure that no grammatically correct sequences were presented, half of the scrambled sequences followed the order “noun – noun – adjective – verb” and the other half followed the order “adjective – verb – noun – noun.”

Every word was played a minimum of 15 and a maximum of 50 times. The sentential and scrambled condition both contained 50 trials with 12 four-word-sequences each resulting in a total of 600 scrambled four-word-sequences and 600 meaningful four-word-sentences. Given task differences between the participant groups, the resultant average time for each task (without breaks) is 29 min for the *passive group*, 33 min for the *word group*, and 16 min for the *sentence group*.

Throughout the experiment, participants were instructed to fixate a white cross at the centre of the screen, to minimise ocular as well as head movements. All stimuli were presented via the MATLAB toolbox Psychtoolbox (Brainard, 1997).

#### Control Analysis

To validate the stimulus material, we performed a control analysis to ensure that the tracking of higher linguistic structures such as phrases or sentences did not reflect tracking of acoustic cues within the acoustic envelope of the material. Therefore, we ran a bootstrap analysis, creating a random set of 50 trials per participant (random selection with replacement; 50 trials corresponding to the number of trials in our study) of the existing EEG and acoustic data per repetition (1000 repetitions). For each repetition, inter-trial-phase-coherence (ITPC) measures were then computed to quantify the strength of tracking at all target frequencies [0.78 Hz (sentences), 1.56 Hz (phrases), and 3.125 Hz (words)] in the auditory and EEG data and then averaged over repetitions and participants. In a Monte Carlo test, these average ITPC values at target frequencies were then compared to the distribution of ITPC values at “chance frequencies,” which were 500 non-harmonic frequencies of the target frequencies. Prior to the Monte Carlo test, the ITPC at these chance frequencies was also averaged over repetitions and participants for acoustic and EEG data. The results of this analysis revealed that only the EEG data showed significant ITPC values at all target frequencies (*p* < 0.001), whereas acoustic data showed a significant ITPC value only at the word rate (*p* < 0.001) but not at the phrase- (*p* = 0.502) or sentence rate (*p* = 0.505). We can therefore conclude that there is no information in the acoustic envelope of the stimulus at either of the higher-level linguistic rates, which could lead to significant peaks in tracking of phrases or sentences by the EEG signal.

### Experimental Design

The experiment included three groups of subjects.

The passive group was naïve to the sentence structure and was instructed to listen passively to the stimulus material.

The word group was also naïve to the sentence structure with their attention being directed to the individual words. After each trial (i.e., 12 four-word-sequences), they judged whether a particular target word which was presented on the screen before each trial, appeared zero, one or two times within the auditory stream of the previous trial. Target words were either adjectives or verbs (50% adjectives and 50% verbs) randomly chosen from the pool of words used in this paradigm. A total of 10% of the trials contained target words.

Both passive and word group were presented with scrambled and sentential word sequences. The sentence group was presented with the sentential condition only, where 10% of the sequences were grammatically incorrect sentences. Participants were informed about the sentential stimulus structure prior to the experiment and were asked to perform a task based on these sentences: they had to identify the grammatically incorrect four-word-sequences (e.g., cold-eat-cell-dog), by responding after each trial (i.e., 12 four-word-sequences) with “yes” or “no.”

For all groups, individual trials were separated by a jittered delay of 1–1.5 s [cf. asynchronous inter-stimulus-interval (aISI) in Figure 1].

### Procedure

During the study, participants sat comfortably in a dim room, ∼50 cm in front of an LCD screen.

The experiment was divided into five blocks. Participants self-initiated a block by pressing a button on the keyboard. They were instructed to take breaks in between blocks if needed. Each block included 20 trials for passive and word group (i.e., sentential and scrambled conditions) and 10 trials for the sentence group (only sentential condition), where each trial consisted of 12 four-word-sequences.

All three participant groups were asked to fixate the central fixation cross throughout the experiment, however, received different task instructions (cf. section “Experimental Design”).

After the experiment, participants of the passive and the word group were asked whether they noticed something specific about the stimulus material without informing them about the sentential structure. This way, we assessed information about whether participants noticed the sentential structure of the stimuli even without any prior knowledge about it. All participants were further debriefed about the study.

### Behavioural Data Analysis

We report median, minimum, and maximum performance for the word and sentence tasks in the respective groups. Participants whose average performance accuracy was not better than chance were excluded from data analysis.

To assess whether the trial type influences performance accuracy for the word group, their performance accuracy was split between sentential and scrambled trials and the averages over these conditions were compared in a paired *t*-test.

### EEG Data Acquisition

EEG data were recorded at 1000 Hz via the software eego64 (ANT Neuro, The Netherlands), using a 124-electrode ANT EEG system (ANT Neuro, The Netherlands) with an extended 10/20 layout. The ground electrode was placed on the left mastoid, whereas the reference electrode was located at CPz. All electrodes showed an impedance of <20 kΩ before the recording started. Individual electrode locations as well as fiducials (nasion, right, and left interauricular points) were recorded prior to the experiment using the software Xensor (ANT Neuro, The Netherlands).

### EEG Pre-processing

EEG data pre-processing was performed using custom-written Matlab scripts (all analysis scripts can be found under the OSF repository following this link: https://osf.io/8pu4a/) and functions of the Matlab toolbox FieldTrip (Oostenveld et al., 2011). EEG data were filtered between 0.01 and 170 Hz, using a FIR filter at filter order 3. Additionally, a notch filter was applied at 48–52, 98–102, and 148–152 Hz using a FIR filter to reduce line noise. Subsequently, the data were epoched into trials starting 1 s before stimulus onset and lasting for the whole length of each auditory stream. This way, trials of 16.36 s were created. Then, data were visually inspected for artefacts as well as noisy channels, which were removed from the data before an ICA was computed (Bell and Sejnowski, 1995) on the down-sampled data (500 Hz), to remove blinks and horizontal eye movements from the data. Finally, noisy channels were interpolated by using data of their neighbours, which were identified via the triangulation method, as implemented in FieldTrip (Oostenveld et al., 2011), before the data were re-referenced to average while reconstructing the reference channel, CPz.

Subsequently, a low-pass filter at 25Hz (butterworth) was applied to the data given the low cut-off of the frequencies of interest (<4 Hz). In preparation for the next analysis step, all trials were further cut to discard the first 2.28 s (resulting in 11 out of the 12 four-word-sequences per trial), which correspond to the 1 s pre-stimulus period and the first four-word-sequence, to avoid including the transient EEG response to the onset of the auditory stimulus (cf. Ding et al., 2017).

### EEG Data Analysis

#### Sensor-Level Analysis

Inter-trial-phase-coherence (ITPC) was used as a measure to quantify whether the brain signal carried signatures of the rhythmic auditory stimulation. This was achieved by first computing the Discrete Fourier Transform (DFT) of the data, for each trial and electrode separately, to transform the signal into the frequency domain with 0.07 Hz resolution [i.e., 1/(15.36 s–1.28 s)]. Equation 1 shows how ITPC was calculated for each frequency (*f*) over all trials (*k*), where *K* is the number of all trials and θ the respective phase angle of the complex-valued Fourier coefficients (cf. Ding et al., 2017). For the passive and the word group, this was done separately for the sentential and the scrambled condition and resulted in 7041 ITPC values for each of the 125 electrodes (i.e., 7041 frequencies × 125 electrodes) per participant and condition.

*ITPC(f) = (*Σ_*k*_*cos(θ_k_))*^2^/*K* + (Σ*_k_ sin(θ_k_))*^2^/*K*

***Equation 1: Inter-trial-phase-coherence (ITPC)***

#### Sensor-Level Statistics

Reported effect sizes and confidence intervals were obtained via the Matlab toolbox MES (“Measures of Effect Size”) by Hentschke and Stüttgen (2011). Outliers were identified via the “boxplot” function of Matlab, as being greater than *q*_3_ + *w* × (*q*_3_ – *q*_1_) or less than *q*_1_ – *w* × (*q*_3_ – *q*_1_), where *w* is the multiplier Whisker and *q*_1_ and *q*_3_ represent the 25th and 75th percentiles of the data, respectively; outliers were marked in the respective figures as filled gray circles. Residual distributions for the performed ANOVAs are included in the Supplementary Material.

##### Average ITPC at Target Frequencies

To test whether each participant group showed significant tracking at the target frequencies, ITPC values were averaged over all electrodes to obtain one average ITPC value per frequency and condition for each participant. Paired *t*-tests were computed for each participant group separately, comparing the ITPC values at one of the target frequencies [word rate (3.125 Hz), phrase rate (1.56 Hz), and sentence rate (0.78 Hz)] with the ITPC values averaged over ±7 surrounding frequencies, which corresponds to ±0.5 Hz (cf. Ding et al., 2017). This way, potential significant peaks (*p* < 0.05) at the target frequencies could be identified. The resulting *p*-values were further corrected within each group and for each condition for multiple comparisons (number of target frequencies) by applying a false discovery rate (FDR) correction (Benjamini and Hochberg, 1995; Yekutieli and Benjamini, 1999).

##### Scalp Distribution Analyses

In order to estimate the scalp distribution of the effects of interest, ITPC values across all electrodes were compared with the cluster mass method of the Matlab toolbox FieldTrip (Oostenveld et al., 2011). Briefly, this involves that adjacent electrodes were grouped in a cluster if their *t*-test *p*-values passed the threshold (detailed below), with the minimum number of electrodes within a cluster set to 4 (adjacent electrodes were identified using the triangulation method). To correct for multiple comparisons, 1000 Monte Carlo permutations of the above method were produced by a randomisation procedure to estimate the probability of the electrode cluster under the null hypothesis (as implemented in the FieldTrip toolbox).

###### ITPC scalp distribution specific for listening to sentences

To investigate the scalp distribution of ITPC specific for listening to sentences, ITPC scalp distributions at the sentence rate (0.78 Hz) were compared between the sentential and the scrambled condition, in a within-subjects design. To that end, we pooled together the data from the passive and the word group. The sentence group was not included, since participants were not exposed to the scrambled condition. Since we expected stronger ITPC at the sentence rate (0.78 Hz) for the sentential compared with the scrambled condition, we applied a one-tailed dependent samples *t*-test at each electrode, and the alpha level and cluster alpha level were set to 0.05.

###### ITPC scalp distribution specific for goal-directed attention to words and sentences

To test for an effect of attention to individual words on ITPC strength, ITPC scalp distributions at the word-frequency (i.e., 3.125 Hz) of the sentential condition were compared in a between-subjects design, between the word and the passive group and between the word and the sentence group, applying two-tailed independent samples *t*-tests at each electrode. The alpha level and cluster alpha level were set to 0.025, as here we tested for both, positive and negative electrode clusters. To investigate the effect of attention to sentences on the tracking strength at the sentence frequency (0.78 Hz), ITPC scalp distributions of the sentence group were compared in a between-subjects design, to those of the sentential condition pooled over participants of the passive and the word group. The alpha level and cluster alpha level were set to 0.025 as here we tested for both positive and negative electrode clusters.

##### ANOVA: Influence of Attention on Tracking Strength at Different Target Frequencies

In order to analyse a potential interaction between attention condition and tracking strength, ITPC values at word (3.125 Hz) and sentence frequency (0.78 Hz) were compared between participants of the word and the sentence group using a two-way between-subjects ANOVA, where attentional manipulation [attention to individual words (word group) vs. attention to whole sentences (sentence group)] and tracking frequency (word- and sentence rate) were the independent variables and ITPC was the dependent variable. Therefore, first, for every participant of the word and sentence group, the 5% of all electrodes showing the highest ITPC values (i.e., 6/125 electrodes) were defined individually for the word (3.125 Hz) and sentence (0.78 Hz) frequency, respectively. Second, the ITPC values were averaged over these electrodes and then served as input for the ANOVA. As 5% is an arbitrary value, we have verified that this choice did not influence the results; similar (and significant) results are obtained when using the ITPC values measured by individual peak electrodes and when using the average ITPC values over the 10% top electrodes.

##### EEG Sensor-Level Bayesian *t*-Tests

To assess evidence supporting the Null hypothesis in the word rate ITPC contrast between word and passive group as well as between word and sentence group, we computed between-subjects Bayesian equivalent two-sample *t*-tests. We therefore computed a Jeffrey–Zellner–Siow Bayes factor (JZS-BF) at each electrode, as implemented in an open-access script^1^. JZS-BF > 3 reflects substantial evidence in support of the tested hypothesis, while JZS-BF < 0.33 reflects substantial evidence in favour of the Null hypothesis.

#### EEG/MRI Co-registration

We recorded the electrode locations of each participant relative to the surface of the head using the infrared camera system device Xensor (ANT Neuro, The Netherlands). Because we did not acquire individual T1-weighted MRI images for all our participants, we used the template files provided by FieldTrip (MRI file, headmodel and grid) and co-registered the standard T1-weighted anatomical scan of the FieldTrip template (1 mm voxel resolution) to the digitised electrode locations using Fieldtrip (Oostenveld et al., 2011).

#### EEG Source Estimation

All source analyses were carried out using Dynamic Imaging of Coherent Sources (DICS; Gross et al., 2001) beamforming. For every participant, a leadfield was computed based on the template grid and headmodel, as well as the individual electrode locations.

##### Source Estimation Specific for Listening to Sentences

To estimate the sources specific for listening to four-word-sentences, we pooled together data of participants of the passive and the word group and compared sentence tracking between the sentential and the scrambled condition, in a within-subjects design.

Therefore, we first computed the cross-spectral density matrix at the sentence frequency (0.78 Hz). We therefore used the method “mtmfft” with a spectral smoothing of ±0.071 Hz and cross-spectral density matrix and power as output, as implemented in FieldTrip. We did that for each trial of the sentential and scrambled condition separately as well as for the combined data (sentential and scrambled trials together). The cross-spectral density matrix of the combined data then served as input to create the common spatial filter for this contrast.

Second, we computed the common spatial filter (regularisation parameter = 5%) which was applied to the cross-spectral-density matrix of the individual conditions. We then contrasted the power of the sentential condition with the power of the scrambled condition in source space, by normalising the difference of the power between the sentential and the scrambled condition by the power of the scrambled condition, and visualised the results on a standard MNI brain using the visualisation software MRIcron^2^.

##### Source Estimation of Attention Effect on ITPC

To investigate the effect of attention on ITPC strength at the sentence frequency on the source level, we compared sentence rate tracking between the sentence and the word group, in a between-subjects design.

Therefore, we first computed the cross-spectral density matrix at the sentence frequency (0.78 Hz) for each trial of the sentential condition of each participant of the sentence and the word group, using the same parameters as described above.

Second, we computed a spatial filter for the sentence tracking for every participant (5% regularisation parameter); since for this contrast, only one experimental condition was investigated per participant, we also computed the noise estimate of the data based on the smallest eigenvalue of the cross-spectral-density matrix, as implemented in Fieldtrip (“projectnoise”). This allowed us to then compute the neural activity index (NAI) by normalising the obtained source results of each participant by the estimated noise obtained from the source analysis. This approach has been shown to circumvent the noise bias toward the center of the brain. Subsequently, the NAI source estimates were contrasted between participants of the sentence and the word group and visualised on a standard MNI brain using the visualisation software MRIcron (see text footnote 2).

## Results

### Behavioural Results

All participants of the word group and 22 out of 24 participants in the sentence group performed the behavioural task above chance (word group: chance level: 33.33%; median performance: 87%, range: 72–96%; sentence group: chance level: 50%; median performance: 80%, range: 45–95%); two participants were therefore excluded from the sentence group based on this criterion (median performance after exclusion of these participants: 81.25%, range: 55–95%). Participants of the word group further showed a benefit of the sentential condition on identifying target words compared with the scrambled condition [T(22) = 7.173; *p* = 3.438 × 10^–7^, effect size (measured as mean difference (md)) = 0.126, ci = [0.089 0.163]; median performance sentential: 94%, range: 78–100%; median performance scrambled: 80%, range: 62–92%]. Furthermore, debriefing of participants of the passive and the word group preceding the experiment revealed that all participants noticed the sentential character of the auditory material of the sentential condition, without any prior instruction.

### EEG Results

Given the ambiguity between the names of the participant groups (i.e., “word group,” “sentence group”) and the rates of cortical tracking (“word rate tracking,” “sentence rate tracking”), we have used in Figures 3–5, labels, carrying the frequency of the respective tracking, rather than only using the descriptive word (i.e., 0.78 Hz; sentences instead of “sentence rate tracking”). These are found in white on black background, in the upper right corner of the respective figure panel.

#### Significant Tracking at All Levels for All Participant Groups

**FIGURE 2:**
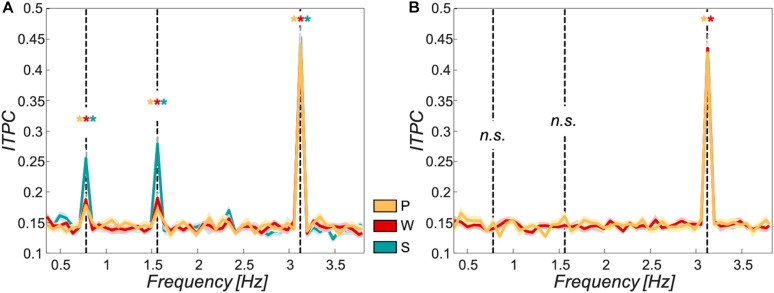
Tracking to target frequencies over different participant groups. Inter-trial-phase coherence was used as a measure of tracking strength to the target frequencies at 0.78 Hz (sentences), 1.56 Hz (phrases), and 3.125 Hz (words). **(A)** sentential condition: All three participant groups (passive group in orange, word group in red, and sentence group in turquoise) showed significant tracking at all target frequencies (*p* < 0.05). **(B)** Scrambled condition: All relevant participant groups (passive group in orange, word group in red; no data shown for sentence group as these participants were only exposed to the sentential condition) showed significant tracking at the word frequency (*p* < 0.05). No significant tracking of phrases or sentences for passive or word group in this condition (*p* > 0.05). Shaded areas around curves show standard error of the mean; asterisks mark significant tracking peaks, “n.s.” reflects non-significant tracking at target frequencies, and black dashed vertical lines mark target frequencies.

The ITPC values showed significant peaks at all target frequencies for each of the participant groups in the sentential condition. The passive and the word group further showed significant ITPC peaks at the word rate for the scrambled condition and no significant peaks for phrase- or sentence rate (see FDR-corrected *p*-values in Table 1). Figure 2 shows ITPC spectra for the passive group in orange (sentential and scrambled conditions), word group in red (sentential and scrambled conditions), and sentence group in turquoise (sentential condition only).

**TABLE 1:** Results of paired *t*-tests quantifying ITPC at target frequencies.

		Sentential condition			Scrambled condition		
		Word	Phrase	Sentence	Word	Phrase	Sentence
**Passive group**	T	10.765	3.061	2.842	9.700	2.101	0.591
	df	19	19	19	19	19	19
	p	9.5 × 10^–9^	0.013	0.016	2.6 × 10^–8^	0.060	0.562
	md	0.299	0.028	0.033	0.281	0.017	0.005
	ci	[0.241 0.357]	[0.009 0.047]	[0.009 0.057]	[0.221 0.342]	[0.0001 0.034]	[-0.012 0.021]
**Word group**	T	13.141	5.359	3.783	11.146	0.544	-1.362
	df	22	22	22	22	22	22
	p	4.1 × 10^–11^	4.4 × 10^–5^	0.002	4.9 × 10^–10^	0.592	0.187
	md	0.295	0.049	0.044	0.289	0.003	-0.006
	ci	[0.249 0.342]	[0.031 0.069]	[0.019 0.067]	[0.236 0.343]	[-0.007 0.012]	[-0.015 0.003]
**Sentence group**	T	11.431	9.233	6.082			
	df	21	21	21			
	p	5.5 × 10^–10^	1.2 × 10^–8^	5 × 10^–6^			
	md	0.315	0.137	0.106			
	ci	[0.258 0.373]	[0.106 0.167]	[0.070 0.143]			

*This table shows for each of the participant groups (passive, word, and sentence group) T-values, degrees of freedom (df), p-values for the paired t-tests comparing ITPC at target frequencies (word, phrase, and sentence rate) with surrounding ±7 frequencies (= ±0.5 Hz), effect size [computed as mean difference (md)], and confidence intervals (ci). Results are shown for sentential and scrambled conditions. P-values were corrected for multiple comparisons over the number of target frequencies for each condition and participant group separately using FDR-correction (Benjamini and Hochberg, 1995; Yekutieli and Benjamini, 1999).*

#### ITPC Spatial Cluster Analysis: Sentential vs. Scrambled Condition

**FIGURE 3:**
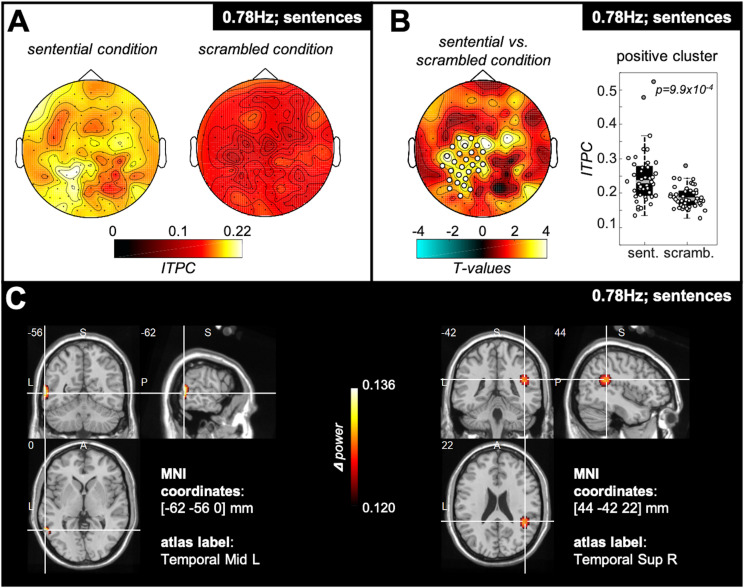
Spatial cluster specific for hearing sentences. **(A)** Topography plots show colour-coded ITPC values at the sentence frequency (i.e., 0.78 Hz) for the sentential and the scrambled condition. The data were pooled over all participants of the passive and the word group. **(B)** When computing the difference between sentential and scrambled condition, a significant positive electrode cluster (*p* = 9.9 × 10^– 4^) was found, located left-lateralised over parieto-temporal recording sites. The box plot to the right reflects ITPC values over the electrodes of the positive cluster, showing stronger ITPC at the sentence frequency for the sentential (“sent.”) compared with the scrambled (“scramb.”) condition. The central gray line marks the median, and the bottom and top edges the 25th and 75th percentiles of the data, respectively. Error bars extend to the extreme values, excluding outliers, and circles represent data of individual participants. Filled gray circles represent outliers. **(C)** Results of the DICS source estimation of the contrast sentential vs. scrambled condition. The source estimation shows colour-coded the difference in power at the sentence frequency between the sentential and the scrambled condition for all subjects of the passive and the word group in functional ortho-plots (functional values are thresholded to present only the top 10% virtual electrodes). The peak areas of the source estimation were identified as left middle temporal gyrus as well as the right superior temporal gyrus. (Letters indicate anatomical landmarks; A = anterior, P = posterior, L = left, S = superior; numbers indicate location of crosshair in the brain in mm).

To test for potential spatial clusters specific to hearing four-word-sentences, we compared ITPC values at the sentence frequency between the sentential and the scrambled condition pooled over participants of the passive group and word group (Figure 3A). We found a significant positive cluster over left-lateralised parieto-temporal recording sites (Figure 3B) showing significantly stronger tracking for the sentential relative to the scrambled condition. DICS source estimates of this contrast reveal peaks in the left middle temporal gyrus as well as in the right superior temporal gyrus (Figure 3C).

#### ITPC Spatial Cluster Analysis: Effect of Attention on Word- and Sentence Rate Tracking

**FIGURE 4:**
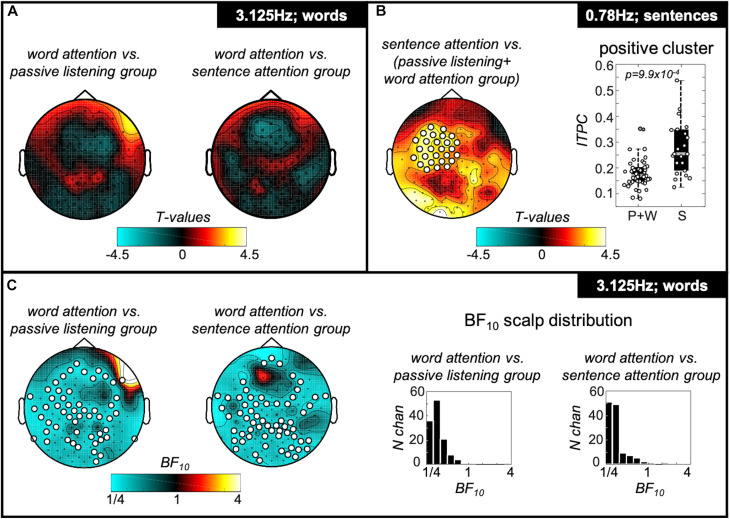
Spatial clusters specific for attentional manipulation. **(A)** Comparison of the word tracking strength between participants who paid attention to individual words (word group) with those participants who did not (passive and sentence group). Topography plots show colour-coded T-values of these comparisons. No significant cluster was found. **(B)** Comparison of the sentence tracking strength between participants who paid attention to individual sentences (sentence group) with those participants who did not (passive and word group). Topography plot shows colour-coded T-values of this comparison. A significant positive cluster was found, located over left-lateralised fronto-temporal recording sites (*p* = 9.9 × 10^−4^). These clusters are further illustrated in the boxplots on the right side of the panel, showing inter-trial-phase-coherence values for participants of passive + word group (“P+W”) and of sentence group (“S”) for the two clusters. The central gray line marks the median, and the bottom and top edges the 25th and 75th percentiles of the data, respectively. Error bars extend to the extreme values, excluding outliers, and circles show data of the individual participants. Filled gray circles represent outliers. **(C)** Results of Bayesian equivalent two-sample *t*-tests. Topographies show that most electrodes in the contrast word vs. passive group as well as word vs. sentence group show substantial evidence in favour of the Null and thus suggest there is no difference in tracking strength at the word rate between these groups. Right side of **C** shows distributions of BF10 over all electrodes on the scalp for both contrasts.

We investigated whether attending to individual words or sentences modulated the tracking strength at the word and sentence frequencies selectively in the sentence condition. Indeed, attending to sentences significantly enhanced tracking at the sentence rate over left-lateralised fronto-temporal recording sites (see Figure 4B). By contrast, goal-directed attention to words did not significantly modulate tracking at the word rate in the sentential condition (Figure 4A). Likewise, Bayes factors provided robust evidence for the absence of an attentional effect on ITPC values at the word-frequency of the sentential condition (i.e., comparable ITPC values for passive group, the word group, and the sentence group). Figure 4C shows topographies of these contrasts where electrodes with a BF10 < 0.33 are marked as white-filled circles and reflect substantial evidence for the Null.

#### Attentional Manipulation Only Shows Effect on Sentence Rate Tracking

**FIGURE 5:**
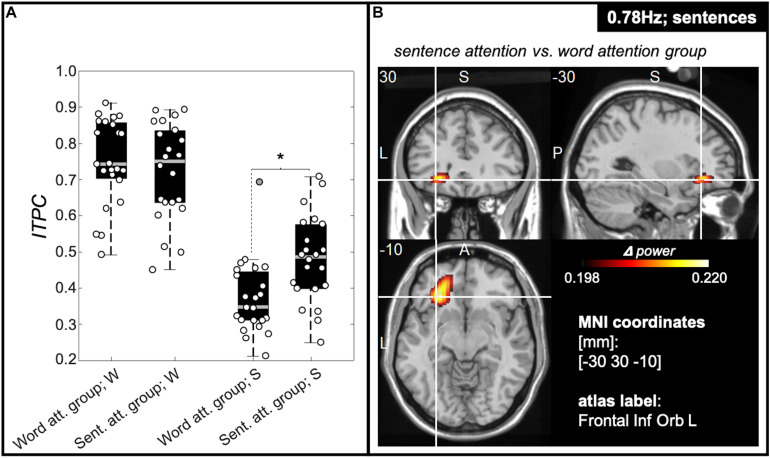
ITPC values at sentence frequency are modulated by attention. **(A)** Box plots representing ITPC values at word- (“W”) and sentence (“S”) frequency for participants of the word group and the sentence group averaged over the 5% of electrodes with the highest ITPC values. We observed a significant interaction between tracking frequency [words (3.125 Hz) and sentences (0.78 Hz)] and attention condition [attention to words (word group), attention to sentences (sentence group)] [*F*(1,91) = 5.92; *p* = 0.017]. *Post hoc t*-tests revealed a significant effect of attention on tracking strength for the sentence rate tracking only, showing significantly stronger ITPC values for the sentence group compared with the word group (see right part of **A)**. Tracking strength at the word rate, however, was comparable between the participant groups (left part of **A**). The central gray lines mark the median, and the bottom and top edges the 25th and 75th percentiles of the data, respectively. Error bars extend to the extreme values, excluding outliers, and circles show data of the individual participants. Filled gray circles represent outliers. **(B)** Source estimation of difference in power at sentence frequency between sentence and word group in functional ortho-plots (functional values are thresholded to present only the top 10% virtual electrodes). The peak region was identified as left inferior frontal gyrus. (Letters indicate anatomical landmarks; A = anterior, P = Posterior, L = left, S = superior; numbers indicate location of crosshair in the brain in mm).

A two-way ANOVA with main factors of attention manipulation (i.e., attention to words; attention to sentences) and target frequencies [3.125 Hz (word rate) and 0.78 Hz (sentence rate)] was computed for the average of 5% of all electrodes showing the highest ITPC values (individually determined for every target frequency and participant). This showed a significant interaction between tracking frequency and attention condition [*F*(1,89) = 7.29; *p* = 0.008; effect size = 0.029]. *Post hoc t*-tests revealed evidence that only tracking at the sentence rate was modulated by the attentional manipulation, showing significantly stronger inter-trial-phase-coherence values at the sentence frequency for the sentence group compared with the word group (*T*(43) = 3.239; *p* = 0.002; effect size (md) = 0.107; confidence interval (ci) = [0.041 0.174]). Inter-trial-phase-coherence values at the word frequency between the word group and the sentence group did not significantly differ [*T*(43) = 0.748; *p* = 0.458; md = 0.028; ci = [-0.048 0.105]; Figure 5A]. Figure 5B shows source estimates of this contrast with a peak in power difference identified over the orbital part of the left inferior frontal gyrus.

## Discussion

Clinical studies of rhythmic language comprehension have identified the potential prognostic value of preserved cortical tracking of higher-level linguistic structures in unresponsive patients (Gui et al., 2020; Sokoliuk et al., 2021). However, which cognitive processes are implicated and necessary to track such higher-level linguistic structures and whether this truly reflects language comprehension is unclear. Given that almost all previous studies using this paradigm in healthy participants have used active tasks based on the sentential structure of the stimuli (see Gui et al., 2020 for a study that used a passive listening condition), it is still unclear to which degree auditory attention is required to track phrases and sentences. Here we provide a more fine-grained characterisation of the functional significance of this cortical tracking that also informs our understanding of the existent clinical data.

All previous studies which used the paradigm in the healthy population informed participants about the sentential stimulus structure and included tasks that required sentence comprehension. One recent study, however, investigated whether high-level tracking would occur without overt attention to sentences, by including a visual distracter task on some of the trials (Gui et al., 2020). While our study shares some aspects with the latter, there are some important differences. In contrast to our study, participants were not naïve to the sentential stimulus structure, and attention was manipulated using a within-subjects design. Moreover, in comparison to our passive participant group, participants’ general attentional state differed as the memory of words and sentences they had listened to was probed after each trial, whereas our passive group did not complete any task. Importantly, the attentional manipulation we used focused on different linguistic structures (i.e., words and sentences) of the same auditory streams and between different participant groups, whereas Gui et al. manipulated attention within the same subjects, toward two auditory streams, the sentential and the scrambled condition. Therefore, this recent study as well as the other previous studies using this paradigm does not allow to dissociate comprehension from attentional sampling of the stimulus material in service of task demands (i.e., “Do these two/four words build a meaningful phrase/sentence?”). The evidence from our study reported here, however, indicates that higher-level cortical tracking is not dependent on the participants completing an active task, as naïve participants who did not perform an active task based on sentences (passive and word group) still showed significant phrase and sentence tracking. This higher-level tracking despite the absence of goal-directed attention could result from implicit attention drawn to the meaningful sentences, which has previously been observed, for instance, in envelope tracking of natural speech stimuli (Kong et al., 2014; Vanthornhout et al., 2019). Nevertheless, we also observed that sentence tracking was significantly enhanced by a task that explicitly required sentence comprehension (i.e., the sentence group). This result is therefore consistent with the role of slower, cross-word neural oscillations in speech comprehension. The fact that tracking of higher-level linguistic structures is present also in the absence of a task is promising for its use in clinical populations who may lack the sustained attentional abilities required to complete a more complex active task, as recently indicated (Gui et al., 2020; Sokoliuk et al., 2021). Moreover, the relatively low cognitive demands required to listen to the stimulus material suggest a good sensitivity of the paradigm, as it is less susceptible to cognitive deficits.

Arguing that an unresponsive patient “comprehends” on the basis of an EEG tracking result is a significant inference in the same way as arguing that a patient is “conscious” on the basis of an EEG data-point. Indeed, in our healthy participant study, here we did not explicitly measure the participants’ comprehension through behavioural means, such as report or subsequent memory tests. Consequently, linking high-level EEG tracking to comprehension requires a balance of evidence across multiple aspects of psycholinguistics. Indeed, the presence of high-level tracking only when participants are awake (Makov et al., 2017) and listening to a language that they understand (Ding et al., 2016) is not sufficient to conclude that tracking is a marker of comprehension, as it is certainly not the case that all speech heard while awake is consciously comprehended. It is also evident that a level of processing of speech is possible without consciousness (see Deacon et al., 2000, although see Kutas and Federmeier, 2000; Lau et al., 2008 for arguments to the contrary). Nevertheless, a recent high-powered study concluded that, while there is evidence for subliminal (unconscious) processing of visually presented individual words, consciousness is required to integrate multiple linguistic units into a sentence (Rabagliati et al., 2018; see also Yang et al., 2017 for similar findings). Consequently, we would conclude that an EEG signal that changes at the boundaries of sentences for which there are no acoustic cues likely reflects a conscious process. Nevertheless, as stated above, without explicit report from the listener, the term “comprehension” may be an inference too far. Indeed, this challenge reflects the more general challenge of inferring consciousness without explicit report in disorders of consciousness (Edlow and Naccache, 2021).

At the source level, the regions specific for sentence comprehension (Figure 3) were identified as left middle temporal gyrus (MTG) and right superior temporal gyrus (STG). These cortical sources have previously been linked to sentence comprehension. For example, the left MTG is more strongly activated when listening to semantically congruent compared with semantically random sentences (Humphries et al., 2007). Furthermore, the right STG has been shown to be activated during semantic processes at the sentence level (Kuperberg et al., 2000). These source estimates are therefore consistent with a functional role of cross-word tracking in comprehending the sentence structure of the stimuli, and replicate findings of the original study introducing this paradigm (Ding et al., 2016), who showed via iEEG evidence for activation of bilateral STG specific for sentence rate tracking.

Our source estimates of the attentional modulation of sentence tracking revealed the orbital part of the left IFG as peak region; left IFG more broadly is a canonical speech comprehension region (e.g., Obleser and Kotz, 2010; Giustolisi et al., 2018; Kroczek et al., 2019). The increased activation within the left IFG in our study in response to increased attention toward sentences could reflect comprehension processes in service of task goals, thus involving regions higher up the language processing hierarchy (left IFG), rather than comprehension itself, which our data indicate is supported by lower-level regions such as left MTG and right STG. While the left IFG has shown stronger activation specific to sentence tracking previously (Ding et al., 2016), it has also been shown to be more strongly activated upon listening to complex compared with simple sentences (Caplan et al., 2000; Walenski et al., 2019). Regarding our findings, even though the sentences were the same between groups and did not differ in complexity, the sentence group focused more on the syntactic structure to identify grammatically incorrect sequences. The level of linguistic processing therefore differed between the word and sentence group, possibly provoking in the latter the stronger activation of the left IFG, reflecting its role in syntactic processing (Tyler et al., 2011; Schell et al., 2017).

Furthermore, our source estimates and scalp distributions showed that increased attention to sentences does not lead to stronger activation of the regions specific for sentence comprehension themselves, but instead to recruitment of higher-level cortex (cf. Figures 3, 5). Previous studies using iEEG found higher-level cortex (left IFG) to be more activated in sentence comprehension contrasts (Ding et al., 2016). However, as already noted, the sentence comprehension contrasts in previous studies included participants who had been instructed about the sentential stimulus structure and who performed a task based on those sentences (see Gui et al., 2020, for a study that used a passive listening condition, followed by a subsequent memory task). In our data, only when comparing sentence rate tracking between participants completing a specific sentence task, and those who do not, did we find increased activation over the left IFG. Together, these results indicate that, through the different foci of attention in our paradigm (words/sentences), we disentangle those regions specifically supporting sentence comprehension (left MTG/right STG) and those involved in comprehension for task goals (i.e., left IFG). In line with our source results is the fact that the scalp distributions of our two effects are markedly different (cf. Figures 3, 4), indicating that not entirely overlapping regions of cortex are implicated in the effects and, therefore, that they reflect dissociable cognitive processes. This spatial dissociation may allow for future investigation of the specific level of cognitive processing performed by an unresponsive patient – i.e., passive comprehension versus active task-directed comprehension. Such separation between patients would be of clinical interest as it could identify patients who are both conscious and capable of following complex verbal commands, albeit covertly. Evidence for this additional command-following ability may then provide a target for active rehabilitation efforts.

Interestingly, goal-directed attention to individual words did not increase word rate ITPC in the word compared with passive and sentence groups. This is surprising as single words here follow a strict rhythm, provoking a steady-state auditory response (Regan, 1989), which, according to existing literature, should be stronger with increased attention toward this acoustic feature (Tiitinen et al., 1993; Bidet-Caulet et al., 2007; Müller et al., 2009; Kim et al., 2011; Roth et al., 2013; Bharadwaj et al., 2014). Potentially, word rate ITPC here does not reflect processing of the word’s meaning, as is required by the task, but rather the participants’ non-semantic expectation to hear individual words at a specific rhythm – an expectation that would be the same for all conditions and groups. Furthermore, as words were monosyllabic, it is not possible to separate tracking of words from tracking of the acoustic envelope. A previous study used disyllabic words to investigate the effect of attention on tracking of individual syllables and words, respectively, and found that tracking of individual syllables did not depend on attention to speech, whereas it was required for tracking of words (Ding et al., 2018). Therefore, we conclude that word rate tracking here likely reflects an acoustic process and that an alternative attention manipulation in future studies focused on the acoustic envelope may allow others to observe attentional modulation of this rhythm. Indeed, evidence for attentional modulation of acoustic envelope tracking has been shown recently with natural speech stimuli (Vanthornhout et al., 2019).

An interesting phenomenon we observed is the ITPC magnitude decrease with increasing linguistic level (words > sentences) (cf. Figure 2). One possible cause could be the frequency with which the different linguistic structures occur in the material. Indeed, words are presented four times as often as sentences. These repetitions could reduce noise in the EEG signal, allowing a more accurate measure of ITPC (Moratti et al., 2007; Vinck et al., 2010; VanRullen, 2016). However, when comparing tracking strength between full sample datasets at the sentence rate with datasets only containing 25% of the trials at the word rate [i.e., corresponding to words (100%) vs. sentences (25%) representation in stimulus material], sentence was still significantly weaker than word tracking [*T*(65) = -17.913; *p* = 1 × 10^–16^]. Therefore, repetition of the linguistic structures (words, sentences) in the stimulus material cannot explain the different tracking strengths at word- and sentence frequency.

Another possibility could be a difference in signal-to-noise ratio (SNR) for tracking of different frequencies. A similar phenomenon has been shown to exist in auditory steady-state responses (ASSR), where certain frequencies lead to a stronger steady-state response than others, showing a sweet spot at around 40 Hz (Purcell et al., 2004; Tichko and Skoe, 2017). This “preference” for certain stimulation frequencies also exists in other modalities, like, for instance, the visual domain, with a maximum steady-state response around 10 Hz (Herrmann, 2001). However, given that the stimulus material used here did not provide any acoustic cues between higher-level linguistic structures such as phrases and sentences, their tracking by the brain signal is of a different nature to classical ASSRs, which are direct bottom-up responses to the acoustic envelope of an auditory signal. While there may be preferential oscillation rates in the auditory system, it could not explain the differences in ITPC magnitude. Possibly, bottom-up signals like the acoustic envelope of the auditory stream, representing the rhythm of individual words, are cleaner and less susceptible to cognitive fluctuations (e.g., effort and distraction) than top-down signals, like the comprehension of phrases and sentences.

Alternatively, the effect could be linked to cognitive effort. Although we showed that sentence tracking does not require goal-directed attention to sentences, it clearly requires participants to be awake (Makov et al., 2017), whereas word rate tracking has been shown to be preserved during sleep. We therefore believe the latter demands less cognitive effort than comprehension of sentences, which could explain the increased ITPC at the word frequency. However, future research needs to investigate this question further to make clear assumptions about the cause of the difference in tracking strength. A potentially interesting point would be to investigate whether this effect is due to the linguistic structure (i.e., whether words always induce stronger ITPC compared with sentences and phrases), or rather to the specific frequency at which these structures occurred (i.e., 3.125 vs. 1.56 and 0.78 Hz). This could be investigated, for instance, by comparing tracking of words and phrases which are played at the same rate (i.e., both at 1.56 Hz).

Our source results provide a potentially valuable insight into the brain regions that should be relatively preserved in patients who show high-level cortical tracking, as well as a means of validating whether any given result is a false positive. Furthermore, our attentional manipulation showed that, even in the absence of goal-directed attention, healthy participants showed high-level cortical tracking. Whilst it was previously shown that this tracking is abolished during sleep (Makov et al., 2017), our results do not allow us to define a minimum level of attention which is required for cortical tracking of higher linguistic structures. Nevertheless, we can conclude that these patients are awake and able to integrate multiple words into a meaningful whole. However, we want to stress that, on the other hand, the lack of significant high-level tracking is no evidence for a patient having no capacity for language comprehension or even for the patient being unconscious. We are aware of the problem of false negatives in this patient group, which could be the result of fluctuations in the level of consciousness of a given patient over the day/week (Wannez et al., 2017; Claassen et al., 2019).

Furthermore, as passive listening is sufficient for higher-level cortical tracking to occur and as we have not explicitly tested language comprehension, we could not necessarily conclude that a given unresponsive patient is having a conscious experience of comprehension of the sentences and phrases. Note, however, as stated above, there is evidence that awareness is required for grouping visually presented individual words into larger linguistic units (Rabagliati et al., 2018). Furthermore, the fact that high-level cortical tracking can be observed during passive listening is also a strength of the paradigm for use in unresponsive patients as it is entirely passive with low cognitive demands, and yet, it provides a valuable insight into the patient’s relative neurocognitive preservation (Edlow and Naccache, 2021). Our results indicate first that sentence/phrase-rate tracking has relatively low cognitive demands and can be elicited during passive listening, and therefore is likely to have good sensitivity in unresponsive patients. Note that we are not claiming to be finding novel evidence of passive listening, but rather are stating that this particular EEG marker of language processing can be detected during passive listening, and therefore is clinically valuable.

Our findings as well as those of previous studies (Ding et al., 2016, 2017; Makov et al., 2017; Gui et al., 2020) suggest that the relationship between high-level linguistic tracking (phrases and sentences) and the recovery of unresponsive patients (Gui et al., 2020; Sokoliuk et al., 2021) is based on the integrity of high-level cortical processing. Previous studies showed that unresponsive patients who show evidence for high-level processing are more likely to recover (Faugeras et al., 2012). This does not impose that those patients have a conscious experience of the speech stimuli; rather, it could reflect preservation of cortical networks which are required for being conscious in the future. Indeed, while the tight link between attention and consciousness could lead us to conclude that an unresponsive patient who exhibits EEG evidence of attentive cortical tracking may be having a subjective experience of comprehension at the time, there are also arguments that attention and consciousness are dissociable (Koch and Tsuchiya, 2007) and therefore that a strong conclusion about the consciousness of the patient should require more active, volitional evidence (e.g., Claassen et al., 2019).

Nevertheless, language comprehension is a key component of assessments of consciousness, and patients with preserved language networks would be more likely to show that they are conscious if asked. While the magnitude of sentence and phrase rate tracking has been shown to predict patient outcome (Sokoliuk et al., 2021), the magnitude of this tracking needs not to reflect quantitative differences in consciousness or comprehension of the listener. Rather, ITPC magnitude may also reflect an average of individually varying fluctuations of arousal and attention across the stimulus.

A limitation of the study is that, besides the attentional manipulation, other cognitive covariables change between participant groups, which could have influenced the results. For instance, while participants are informed about the target word identity before each word-group trial, the participants of the sentence group have to be attentive until the grammatically incorrect four-word-sequence is presented, thus potentially varying cognitive effort across these groups. This may also lead to a difference in expectation, since the word group is provided with a clear top-down goal (i.e., the target word), compared to the sentence group. Furthermore, the chance levels differ between sentence and word group (50 vs. 33%). Indeed, task performance was significantly lower [*T*(43) = 2.250; *p* = 0.030] for participants of the sentence group, compared with the word group. Follow-up studies may seek to match these covariables by, for example, titrating individual participant performance. Another aspect in which the sentence group differs from the two other groups is that sentential and scrambled trials were presented in a random order for the word and passive group, whereas the participants of the sentence group were only presented with sentential trials. This could have provoked a difference in ITPC at the sentence and phrase rate. We therefore performed a control analysis comparing for the word and passive group the different trial types for the sentential condition. Specifically, we separated sentential trials which were preceded by a scrambled trial from those which were preceded by a sentential trial and computed ITPC separately for those trial types. There were no significant differences in ITPC for either the passive group [phrase rate: *T*(19) = 0.948; *p* = 0.355; sentence rate: *T*(19) = 0.172; *p* = 0.514], or for the word group [phrase rate: *T*(22) = -0.055; *p* = 0.957; sentence rate: *T*(22) = 0.091; *p* = 0.928]. We can therefore conclude that the difference in higher-level ITPC between the sentence group and the two other participant groups does not result from the presentation order of sentential and scrambled trials.

Future studies might elucidate further whether high-level cortical tracking truly reflects language comprehension. Although testing this by probing participants’ memory could be problematic, since a lack of memorised sentences would not necessarily presume a lack of comprehension while participants were listening to the sentences.

Another limitation of this study is the lack of individual headmodels in the source analysis, which, together with the rather low spatial resolution of EEG data, can lead to decreased accuracy of the results if compared with other methods, like, for instance, MEG. Therefore, source level results should be interpreted with caution.

## Conclusion

Here we replicated (Ding et al., 2016, 2017) and extended previous findings by characterising the role of auditory attention on cortical tracking of speech stimuli. We observed that tracking of higher linguistic structures does not depend on and therefore cannot be solely explained by goal-directed attention to sentences. However, goal-directed attention to sentences did significantly increase sentence tracking in higher-level cortex, consistent with a neural enhancement in service of task demands. The low attentional effort required for sentence tracking (and comprehension) in this paradigm potentially reflects the importance of speech in humans and underlines the advantage for its application in clinical assessment of unresponsive patients.

## Data Availability Statement

The datasets presented in this study can be found in online repositories. The names of the repository/repositories and accession number(s) can be found below: Open Science Framework: https://osf.io/8pu4a/.

## Ethics Statement

The studies involving human participants were reviewed and approved by the Ethical Review Committee of the University of Birmingham. The participants provided their written informed consent to participate in this study.

## Author Contributions

RS, LM, UN, and DC contributed to the conception and design of the study. RS, GD, and DC contributed to the acquisition and analysis of data. RS and DC contributed to drafting the text and preparing the figures. All authors contributed to the article and approved the submitted version.

## Conflict of Interest

The authors declare that the research was conducted in the absence of any commercial or financial relationships that could be construed as a potential conflict of interest.

## Publisher’s Note

All claims expressed in this article are solely those of the authors and do not necessarily represent those of their affiliated organizations, or those of the publisher, the editors and the reviewers. Any product that may be evaluated in this article, or claim that may be made by its manufacturer, is not guaranteed or endorsed by the publisher.

## Footnotes

1
https://github.com/anne-urai/Tools/tree/master/stats/BayesFactors

2
www.nitrc.org
