# Supplementary material for: The Influence of Auditory Attention on Rhythmic Speech Tracking: Implications for Studies of Unresponsive Patients
Source: Front Hum Neurosci. 2021 Aug 11;15:702768. doi: 10.3389/fnhum.2021.702768 (PMC8385206; doi:10.3389/fnhum.2021.702768)

Supplementary material

1. Effect of attentional modulation for word and sentence tracking between participant groups

In addition to the ANOVA analysis from the main text, where the effect of attention on word and sentence rate tracking was analysed between the sentence and the word participant groups (see Figure 5A), we have repeated this analysis also including the passive participant group. This group was not included in the main analysis as its cognitive state is not directly comparable with the other participant groups who were only passively listening to the stimuli. The ANOVA was performed for three datasets: i) averaging word and sentence rate tracking values per participant over the top 10% electrodes, ii) the top 5% electrodes and iii) using the tracking values of one individual peak electrode only.

The results of all three ANOVA analyses are comparable with the results presented in the main text, when comparing only sentence and word group. Hence, we observed a significant interaction between tracking frequency and attention condition / participant group (10% top electrodes: $F(2,129) = 5.69$; $p=0.004$; 5% top electrodes: $F(2,129) = 5.69$; $p=0.004$; peak electrode: $F(2,129) = 6.34$; $p=0.002$). Like previously, post-hoc t-tests revealed evidence that only tracking at the sentence rate was modulated by the attentional manipulation, showing significantly stronger sentence rate tracking for the sentence group compared with both the word group (sentence level tracking, sentence vs. word group: 10% top electrodes: $T(43) = 3.434$; $p = 0.001$; 5% top electrodes: $T(43) = 3.239$; $p=0.002$; peak electrode: $T(43) = 2.784$; $p = 0.008$) and the passive group (sentence level tracking, sentence vs. passive group: 10% top electrodes: $T(40) = 3.929$; $p = 3.283 \times 10^{-4}$; 5% top electrodes: $T(40) = 3.840$; $p=4.287 \times 10^{-4}$; peak electrode: $T(40) = 3.705$; $p = 6.384 \times 10^{-4}$). Like shown previously, ITPC values at the word frequency did not significantly differ between the word group and the sentence group (word level tracking, word vs. sentence group: 10% top electrodes: $T(43) = 0.544$; $p = 0.589$; 5% top electrodes: $T(43) = 0.748$; $p=0.458$; peak electrode: $T(43) = 0.880$; $p = 0.384$), neither did ITPC values at the word rate differ between word and passive group (word level tracking, word vs. passive group: 10% top electrodes: $T(41) = 0.194$; $p = 0.847$; 5% top electrodes: $T(41) = 0.192$; $p=0.849$; peak electrode: $T(41) = -0.116$; $p = 0.908$).

2. Residual distributions of ANOVA analysis

a) Considering the average ITPC value over the top 5% electrodes

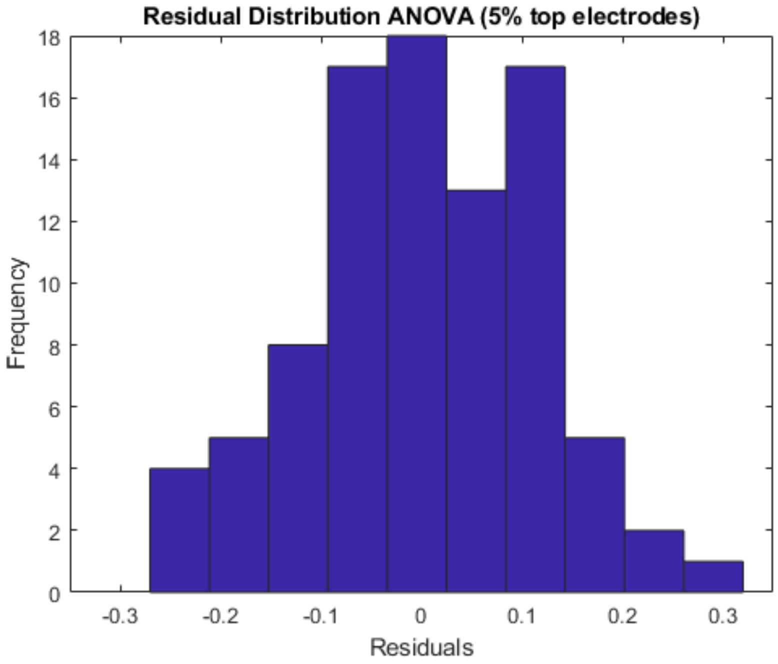

b) Considering the average ITPC value over the top 10% electrodes

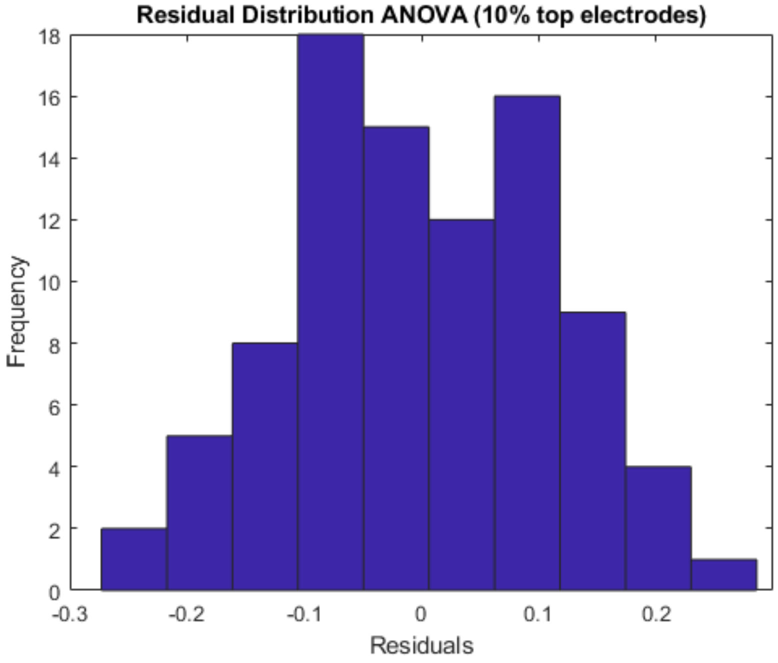

c) Considering the ITPC value of the peak electrode

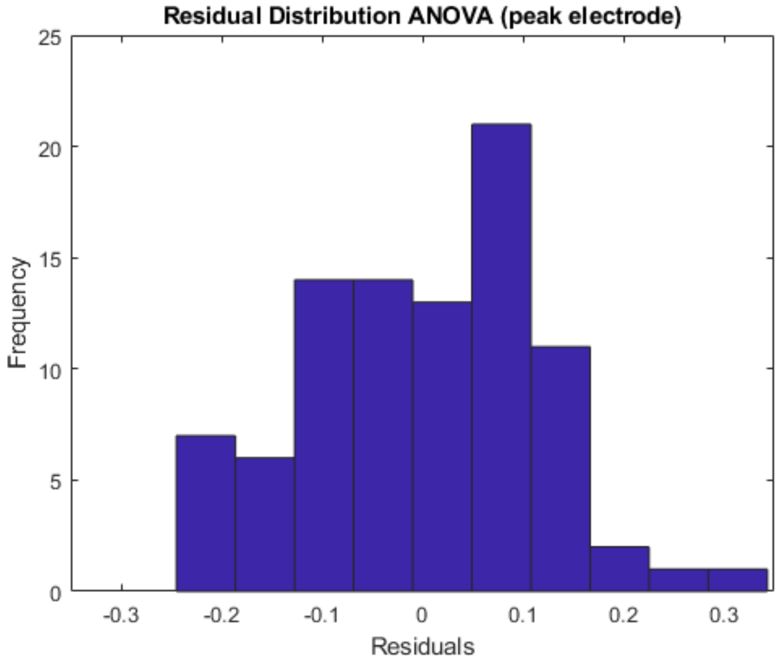

Supplement: Supplementary file 1 [file Data_Sheet_1.pdf]
